# Numerical Investigation of a Designed-Inlet Optofluidic Beam Splitter for Split-Angle and Transmission Improvement

**DOI:** 10.3390/mi12101200

**Published:** 2021-09-30

**Authors:** Ting-Yuan Lin, Chih-Yang Wu

**Affiliations:** Department of Mechanical Engineering, National Cheng Kung University, Tainan 701401, Taiwan; tuba0306@hotmail.com

**Keywords:** optofluidics, beam splitter, gradient refractive index, transmission, ray tracing

## Abstract

The beam splitter is one of the important elements in optical waveguide circuits. To improve the performance of an optofluidic beam splitter, a microchannel including a two-stage main channel with divergent side walls and two pairs of inlet channels is proposed. Besides, the height of the inlets injected with cladding fluid is set to be less than the height of other parts of the microchannel. When we inject calcium chloride solution (cladding fluid) and deionized water (core fluid) into the inlet channels, the gradient refractive index (GRIN) developed in fluids flowing through the microchannel splits the incident light beam into two beams with a larger split angle. Moreover, the designed inlets yield a GRIN distribution which increases the light collected around the middle horizontal line on the objective plane, and so enhances the transmission efficiency of the device. To demonstrate the performance of the proposed beam splitter, we use polydimethylsiloxane to fabricate the microchannel. The results obtained by simulation and experiment are compared to show the effectiveness of the device and the validity of numerical simulation. The influence of the microchannel geometry and the flow rate ratio on the performance of the proposed beam splitter is investigated.

## 1. Introduction

Optofluidics aims at the fusion of integrated optics with microfluidics [1]. A wide variety of applications inspired by optofluidics have been developed for solar energy collection [2], water treatment [3], chemical and biological detection and analysis [4,5], sensors [6] and tunable optical devices [7,8]. Optofluidic devices have several advantages over traditional optical devices, including compactness, tunability, reconfigurability and integration. Moreover, the advancements of microfabrication have enabled the realization of some optofluidic devices to be integrated as part of lab-on-a-chip (LOC). Therefore, optofluidics and its applications have earned much attention over the past two decades.

A large number of optofluidic devices have been proposed and investigated, such as microlenses, prisms, waveguides and beam splitters. Seow et al. [9] reported a tunable liquid microlens by controlling the flow rates of the three injected flows with different refractive indices into an expansion chamber. Shi et al. [10] proposed an optofluidic microlens using a static polydimethylsiloxane (PDMS) lens and an air-water interface that can be reshaped to tune the focal point of incident light. Mao et al. [11] reported the design and characterization of a tunable microlens generated by the interface between two miscible fluids of different refractive indices flowing through a 90-degree curve in a microchannel. Mao et al. [12] proposed an optofluidic microlens with liquid gradient refractive index (L-GRIN) to focus light. Mishra et al. [13] presented a liquid-liquid interface microlens formed by two mutually immiscible fluids. By adjusting the hydrostatic pressures and electric field to control the curvature of the interface, the focal length and spherical aberration can be tuned. Le et al. [14] designed an in-plane L-GRIN microlens that realized two-dimensional light beam focusing dynamically. Miccio et al. [15] have shown that a red blood cell can serve as an adaptive optofluidic microlens, which can be used in the application of blood diagnosis. Hamilton et al. [16] developed a three-dimensional focusing device, which was integrated with optical waveguides and used to analyze fluorescent signals from beads in fluid flow. A prism can also be formed by injecting different fluids with different flow rates to a chamber to manipulate light [17,18]. Wolfe et al. proposed an L-GRIN optical waveguide [19] and an optical splitter based on liquid-liquid waveguide [20]. Moreover, with liquids containing different dyes, the device [20] can also be used as a filter. Schmidt and Hawkins presented literature reviews on liquid-core optical waveguides with emphasis on suitability for creating fully planar optofluidic LOC [21] and their fabrication methods and common structures [22]. Yang et al. [23] presented an optofluidic waveguide for light bending and manipulation by pumping two fluids through a straight microchannel, where a bidirectional GRIN-like profile with a higher refractive index in the core region can be achieved at a relatively low flow rate. Yang et al. [24] reported a waveguide with the designed GRIN achieved by the convection-diffusion process of liquid flowing streams with a lower refractive index in the core stream to split a light beam into two parts with a large split angle. It has also been demonstrated the asymmetrical split angle can be achieved by varying the concentration of the cladding flow. Li et al. [25] demonstrated realization of a tunable optofluidic Y-branch waveguides in a planar microchannel by introducing Dean flow.

Waveguides are critical components in optical systems and the optical Y-branch splitter is one of the most important elements in optical waveguide circuits. Yang et al. [24] have shown that a large tunable split angle up to 30 degrees can be achieved by tuning the flow rates of the streams flowing through the inlets of a microchannel based on the designed GRIN achieved by the convection-diffusion process of liquid flowing streams with a lower refractive index in the core stream. In light of Yang’s design [24], we try to explore a little further into microchannel structure which yields the GRIN for the optical splitter with a larger split angle and better transmission efficiency. A designed microchannel, including a divergent main channel, two pairs of inlet channels injected with core and cladding fluids and a pair of outlets, is proposed to enlarge the split angle. The L-GRIN beam splitter may enhance the transmission efficiency of the device by setting the height of the inlets injected with cladding fluid to be less than the height of other parts of the microchannel. To demonstrate the performance of the proposed beam splitter, we use PDMS to fabricate the beam splitter. The results obtained by numerical simulation and experiment are compared to show the effectiveness of the proposed device and the validity of numerical simulation. The influence of the geometry parameters of the microchannel and the flow rate ratio of the two injected fluids on the performance of the L-GRIN beam splitter is investigated.

## 2. GRIN Beam Splitter Design

**Figure 1:**
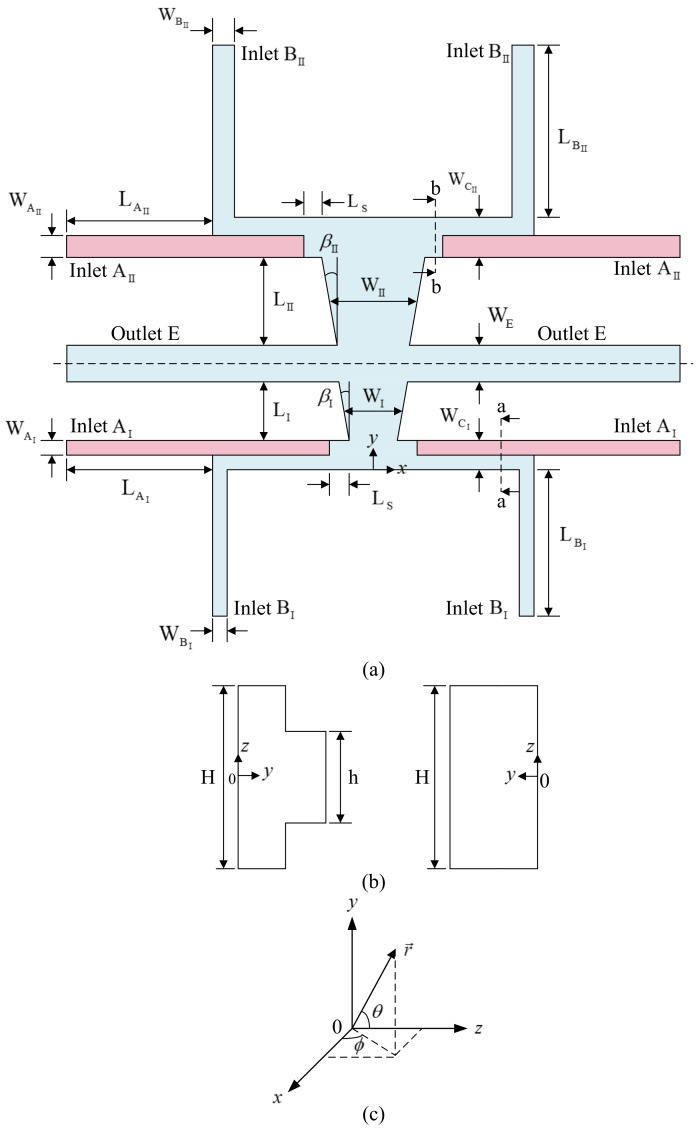
(**a**) Top view of the proposed microchannel; the height of the channels in pink is h and the height of the channels in light blue is H; (**b**) Section a-a (left) and section b-b (right); (**c**) The coordinates.

To improve the performance of an L-GRIN beam splitter, we adopt a two-stage main channel with symmetric inlet and outlet microchannels and set the sidewalls of each stage of main microchannel to be divergent, as shown in Figure 1. The main channel can be viewed as two parts by taking the centerline of the outlets as the baseline. The primary flow in the first part of the two-stage microchannel is in the direction to the positive *y*-axis, while that in the second one is in the direction to the negative *y*-axis. There are two pairs of inlets, $A_{I}$, $B_{I}$, $A_{\mathrm{II}}$ and $B_{\mathrm{II}}$, and the outlets of the two parts are merged together, as shown in Figure 1a. Here, we use A and B to represent the inlets injected with calcium chloride (CaCl_2_) solution (cladding fluid) and the inlets injected with deionized water (core fluid), respectively, and use E to represent the outlets. The inlets $A_{I}$ and $B_{I}$ are merged together before entering the main channel; we use $C_{I}$ to represent the merged inlet channel. Similarly, we use $C_{\mathrm{II}}$ to represent the merged inlet channel of $A_{\mathrm{II}}$ and $B_{\mathrm{II}}$. Thus, the widths of the inlets are denoted by $W_{A_{I}}$, $W_{B_{I}}$, $W_{C_{I}}$, $W_{A_{\mathrm{II}}}$, $W_{B_{\mathrm{II}}}$ and $W_{C_{\mathrm{II}}}$, and the width of the outlets is denoted by $W_{E}$. The subscripts I and II in the symbols used denote the first and the second part of the microchannel, respectively, for convenience. The symbol $W_{I}$ denotes the width of main channel of the first part at the location $L_{I}/2$ away from the outlet microchannel and we set $W_{I}=2W_{C_{I}}$. Similarly, the width of the second part of main channel at the location $L_{\mathrm{II}}/2$ away from the outlet microchannel is denoted by $W_{\mathrm{II}}$ and we set $W_{\mathrm{II}}=2W_{C_{\mathrm{II}}}$.

To enhance the transmission efficiency of the device, we set the height of the first part of inlets $A_{I}$ and $A_{\mathrm{II}}$, denoted by h, to be less than the height of other parts of the microchannel, denoted by H, and the height of inlets $A_{I}$ and $A_{\mathrm{II}}$ is enlarged to H at the locations $L_{S}$ away from the main channel. In Figure 1a, the height of the channels in pink is h and the height of the channels in light blue is H. Cross sections of an inlet branch at the locations with distance from the main channel greater and less than $L_{S}$ are shown in the left and the right of Figure 1b, respectively. In contrast to the bidirectional GRIN achieved by the convection-diffusion process in the beam splitter with uniform channel height proposed by Yang et al. [24], the GRIN distribution achieved by the convection-diffusion process of liquid flowing streams with a lower-refractive-index core stream in the present device with deliberate variation of inlet channel height is three-dimensional (3D). The 3D GRIN, except splits an incident light coupled into the device into two parts, makes more light arrive a zone around the middle horizontal line of an objective plane, where the output optical fibers are coupled to the device. Thus, the 3D GRIN may enhance the transmission efficiency of the present device. The effect of the 3D GRIN generated in the present device on beam splitting will be demonstrated and discussed later.

When we remove the second part of the microchannel, adopt the main microchannel with the side walls parallel to the *y*-axis, and set the height of inlets (h) to be equal to the height of the main microchannel (H), the present design reduces to a one-stage beam splitter similar to the L-GRIN beam splitter proposed by Yang et al. [24].

There are a very large number of geometrical parameters influencing the performance of the proposed beam splitter. The present work focuses on the effects of geometric modification of the inlets injected with cladding fluid and the two-stage main channel with divergent side walls on the performance of the proposed beam splitter. The former is represented by the ratio of the height of inlets $A_{I}$ and $A_{\mathrm{II}}$ to the main-channel height, h/H. The latter is described by the lengths of the two parts of the main channel ($L_{I}$ and $L_{\mathrm{II}}$) and the divergent angles of the side wall of the two parts of the main channel ($\beta_{I}$ and $\beta_{\mathrm{II}}$).

## 3. Numerical Simulations and Geometrical Parameter Analysis

The parameters influencing the performance of the beam splitter considered include h/H, $L_{I}$, $L_{\mathrm{II}}$, $\beta_{I}$, $\beta_{\mathrm{II}}$ and the flow rate ratio of the injected cladding fluid and the injected core fluid. To select the values of those parameters enhancing the performance of beam splitter, we first use numerical simulation to investigate the influence of every single parameter. Then, the efficiency and effectiveness of beam splitters with different combinations of the above parameters are examined. The GRIN distribution formed by the diffusion between the two selected working fluids, calcium chloride solution and deionized water, determines the paths of light in the L-GRIN device. Thus, the simulation includes the convection-diffusion in the flowing streams and the ray tracing of light beam in the device. Here, we use Computational Fluid Dynamics (CFD) software for the former and self-developed C++ codes for the latter, respectively.

The convection-diffusion process of flowing streams with calcium chloride solution in the cladding stream is described by the continuity equation, the momentum equation and the species convection-diffusion equation for steady flows expressed as

(1) $$\nabla \cdot \left(\rho \vec{v}\right)=0$$

(2) $$\nabla \cdot \left(\rho \vec{v}\vec{v}\right)=-\nabla p+\nabla \cdot \left[\mu \left(\nabla \vec{v}+\nabla {\vec{v}}^{T}\right)+\nabla \left(-\frac{2\mu }{3}\nabla \cdot \vec{v}\right)\right]$$

(3) $$\nabla \cdot \left(\rho \vec{v}c_{A}\right)=\nabla \cdot \left(\rho D_{\mathrm{AB}}\nabla c_{A}\right)$$

where $\vec{v}$ is the fluid velocity, in m/s, ρ the fluid density, in kg/$m^{3}$, p the pressure, in ${N/m}^{2}$, µ the fluid dynamic viscosity, in kg/m⋅s, $D_{\mathrm{AB}}$ the diffusion coefficient of fluid A in fluid B, in $m^{2}/s$, and $c_{A}$ the mass fraction of calcium chloride solution.

In this work, we use the curve-fitting method to construct the relationship between the normalized mass fraction of calcium chloride solution and the fluid density [26]. The result can be expressed as

(4) $$\rho =-17.96\;\Phi_{A}^{4}+42.08\;\Phi_{A}^{3}+11.15\;\Phi_{A}^{2}+248.05\;\Phi_{A}+998.24$$

where the normalized mass fraction of calcium chloride solution is defined as

(5) $$\Phi_{A}=c_{A}/c_{A,0}$$

with $c_{A,0}$ denoting the inlet mass fraction of calcium chloride solution. Similarly, the relation between the normalized mass fraction of calcium chloride solution and the fluid viscosity [26] can be expressed as

(6) $$\mu =0.002992\;\Phi_{A}^{4}-0.003044\;\Phi_{A}^{3}+0.001993\;\Phi_{A}^{2}+0.000506\;\Phi_{A}+0.001009.$$

The diffusion coefficient of calcium chloride solution with a mass fraction ($c_{A}$) from 0 to 0.3 in deionized water is approximately from $1.153\times 10^{-9}$ to $1.31\times 10^{-9}{\;m}^{2}/s$ [27]. Here, we take the average value of $1.229\times 10^{-9}{\;m}^{2}/s$ in the simulation.

Calcium chloride solution (fluid A) with $c_{A}=c_{A,0}=0.3$, ρ=1281.6 ${\mathrm{kg}/m}^{3}$ and μ= 0.003467 kg/m⋅s at $20{\;}^{\circ }C$ is injected at inlets $A_{I}$ and $A_{\mathrm{II}}$, and deionized water (fluid B) with $c_{A}=0$, ρ= 998.2 ${\mathrm{kg}/m}^{3}$ and μ= 0.001003 kg/m⋅s at $20{\;}^{\circ }C$ is injected at inlets $B_{I}$ and $B_{\mathrm{II}}$. The no-slip condition is imposed on all solid walls. The lengths of inlets are set to be ten times of hydraulic diameters based on their cross section and the outlet pressure is set to be atmospheric pressure. The solid walls are set to be impermeable.

In this work, we use the CFD software, ANSYS Fluent 15.0 (ANSYS, Inc., Canonsburg, PA, USA), to simulate the flow field and the concentration distribution by solving the aforementioned governing equations. The governing equations are discretized by using finite volume method. The coupled problems of pressure and velocity in the momentum equation are solved by using Semi-Implicit Method for Pressure Linked Equations (SIMPLE). The convective terms are handled by using second-order upwind method. During the calculation, the residual at each cell is calculated and normalized by dividing the largest absolute value of the residual in the first five iterations. When the normalized residual is less than $10^{-4}$, the solution is said to be converged.

After the calculation, we use the CFD Post of ANSYS Fluent to visualize the flow field and export the mass fraction data at every single node which can be converted into the refractive index data. Again, we use curve-fitting method to obtain the relationship between the normalized mass fraction of calcium chloride solution and the refractive index based on the datasheet [26]. The refractive index can be expressed in terms of $\Phi_{A}(x,y,z)$ as

(7) $$n=1.332+0.079\;\Phi_{A}$$

Thus, we can obtain the refractive index at every grid point, where the value of $\Phi_{A}$ is available.

In this work, the selected working fluid is the mixture of deionized water (n = 1.33) and calcium chloride solution with a mass fraction of 30% (n = 1.41) and the device is composed by two PDMS layers (*n* = 1.412). The PDMS has good transparency at visible wavelengths with very low absorption loss. Different L-GRIN distributions can be achieved by adjusting the flow rates of both fluids injected into the microchannel of the optofluidic beam splitter.

**Figure 2:**
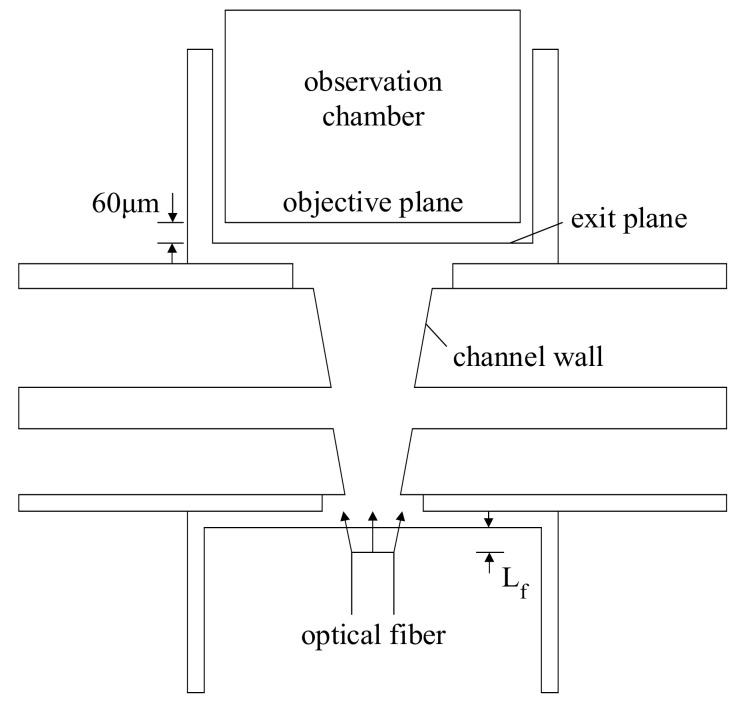
Schematic of the set-up including the microchannel, the optical fiber and the observation chamber for image acquisition.

When an incident beam from an optical fiber enters the proposed device, it will be refracted continuously by the GRIN medium. To examine the refracted light propagation, we develop ray-tracing codes for the set-up schematically shown in Figure 2. The light source, a green laser with a wavelength of λ=532 nm, is coupled with an optical fiber which is then set into PDMS layers. The distance between the output end of the optical fiber and the microchannel is $L_{f}$, and the central lines of the optical fiber and the microchannel are collinear. Moreover, an observation chamber with a vertical wall located at a distance of 60 μm from the side wall of the inlet $B_{\mathrm{II}}$ is also fabricated in the set-up, as shown in Figure 2. Here, we use multimode optical fiber as the coupled fiber. The wavelength range of the optical fiber adopted is from 250 to 1100 nm with a refractive index distribution of step index, and the inner and outer diameters of the optical fiber are 100 and 140 μm, respectively. Numerical aperture ($N_{A}$) of the optical fiber can be expressed as [28]

(8) $$N_{A}=\sqrt{n_{1}^{2}-n_{2}^{2}}=n\;\sin\theta_{A}$$

where $n_{1}$ is the refractive index of the core, $n_{2}$ is the refractive index of the clad, n is the refractive index of surroundings where the optical fiber is set, and $\theta_{A}$ is the half acceptance angle. Since the optical fiber is set in PDMS layers, its numerical aperture is estimated to be 0.16, which leads to a maximum divergent angle of 9 degrees [11]. The greater $L_{f}$ is, the greater the radius of the beam is at the location, where divergent light enters the microchannel. In this work, $L_{f}$ is set to be 50 μm and the radius of the beam at the plane where the rays enter the microchannel is denoted by $r_{0}$.

The point of incidence where a ray enters the microchannel on the plane (y = 0) can be written as ($x_{i}$, 0, $z_{i}$) with $x_{i}$ and $z_{i}$ denoting the x and z coordinates of the point of incidence, respectively. To simulate the propagation of the light from an optical fiber, we treat the light energy leaving the outlet of the optical fiber as a bundle belonging to a set of $N_{b}$ bundles of equal energy; the path of the bundle is the same as a ray leaving the optical fiber. On the plane y = 0, the initial position of a ray, ($x_{i}$, $z_{i}$), can be expressed as

(9) $$x_{i}=r_{0}R_{x},$$

(10) $$z_{i}=r_{0}R_{z},$$

where $R_{x}$ and $R_{z}$ are random numbers uniformly distributed between 0 and 1, and $r_{i}=\sqrt{x_{i}^{2}+z_{i}^{2}}\leq r_{0}$. On account of the divergent angle of the incident beam, we assume that the relationship between incident angle and initial position can be expressed as

(11) $$\theta_{i}=\theta_{\max}\;\left(\frac{r_{i}}{r_{0}}\right)\;\left(\frac{\pi }{180}\right)$$

(12) $$\phi_{i}=\tan^{-1}\left(\frac{x_{i}}{z_{i}}\right),$$

where $\theta_{\max}$ is the maximum divergent angle determined by numerical aperture.

While assuming geometrical optics holds, the path of rays in the GRIN medium can be obtained by solving the ray equation of geometric optics [29]:

(13) $$\frac{d}{ds}\left[n\left(\vec{r}\right)\frac{d\vec{r}}{ds}\right]=\nabla n\left(\vec{r}\right),$$

where $\vec{r}=x\hat{i}+y\hat{j}+z\hat{k}$ is the position vector defining a point on the ray, $n\left(\vec{r}\right)$ the refractive index at a position $\vec{r}$ on the trajectory and *s* a path length along the trajectory. Nonetheless, the above equation is not in an appropriate form for numerical integration. Employing the variable *t* defined as dt=ds/n [30], the ray equation can be expressed as

(14) $$\frac{d\vec{r}}{dt}=\vec{T},$$

(15) $$\frac{d\vec{T}}{dt}=n\left(\vec{r}\right)\nabla n\left(\vec{r}\right),$$

where

(16) $$\vec{T}=n\frac{d\vec{r}}{ds}.$$

Equations (14) and (15) are a system of ordinary differential equations and can be solved with the initial condition that an optical ray vector $\vec{T}$ is known and s = 0 at $\vec{r}={\vec{r}}_{i}$ to compute the ray trajectory. Here, we use Runge-Kutta Dormand-Prince (RKDP) method [31] to solve Equations (14) and (15) with a given refractive index distribution. We refer to the series of points obtained in the tracing procedure as the Runge-Kutta points.

While tracing the ray trajectories by the RKDP scheme, refractive-index values and gradients should be given at the Runge-Kutta points. Thus, during the tracing procedure, the refractive-index value and gradient of each Runge-Kutta point have to be retreived from the refractive indices at grid points obtained by the CFD software and Equation (7). Here, we use Moving Least Squares (MLS) method [32,33] to reconstruct the refractive index at the Runge-Kutta points.

When the ray intersects with a surface, the position solved by the RKDP method is usually not exactly at the surface. We use the method based on cubic spline interpolation [34] to determine the ray-surface intersection point. Once we have the coordinates of the ray-surface intersection point, we can calculate the directions of a ray after either reflection or refraction by using the law of reflection or the Snell’s law [29].

The description of light propagation shall include the ray trajectories as well as the energy carried by the bundles. When a ray intersects the interface between the working fluid and the PDMS, the reflectivity (ρ) is obtained by Fresnel’s relation [29] from the directions of ray and the refractive indices of the media on both sides of the interface. The transmissivity τ at the intersection point equals 1 − ρ. Besides, the energy absorbed by the mixture and the PDMS is assumed to be negligible, and so the energy carried by a bundle only varies when a ray intersects the interface.

To evaluate the performance of the proposed beam splitter, we first calculate the amount of energy carried by bundles arriving the exit plane and the objective planes shown in Figure 2, and then calculate the beam split angle. Here, the amount of energy calculated is normalized with respect to the total energy of bundles leaving the optical fiber. The normalized energy arriving the region located between z=50 μm and −50 μm on the objective plane is denoted by $E_{\pm 50\mu m}$. Besides, we set a circle with a radius of 50 μm on the objective plane at $y=y_{1}$ and calculate the energy gathered inside the circle with its center located at varying *x* on the positive *x*-axis. The energy gathered inside the circle is equivalent to the energy arriving an optical fiber coupled with the L-GRIN beam splitter and the radius of the circle is equal to the radius of the optical fiber. Similarly, we normalize the energy gathered inside the circle and denote the normalized energy as $E_{ob}(x)$. When the maximum value of $E_{ob}$ is found in a circle with its center at a location $\left(x_{1},\;y_{1},\;0\right)$, the circle is said to be the objective circle and the center of the circle is treated as the center of one of the split beams on the objective plane. Similarly, we can carry out the calculation of the $E_{ob}$ for the circles on the exit plane at $y=y_{2}$ to find the center of the split beam, $\left(x_{2},\;y_{2},\;0\right)$, on the exit plane. Once we determine $\left(x_{1},\;y_{1},\;0\right)$ and $\left(x_{2},\;y_{2},\;0\right)$, the split angle can be calculated as

(17) $$\theta_{s}=2\tan^{-1}\left(\frac{x_{1}-x_{2}}{y_{1}-y_{2}}\right)$$

## 4. Fabrication and Experiment Apparatus

The microchannel of the proposed beam splitter is symmetric to the middle horizontal plane (z=0). Thus, the device is constructed by bonding two identical haves of the microchannel. Each half of the microchannel is equipped with two dimensions in height, h/2 and H/2. A master mold of SU-8 (50) film (MicroChem, Round Rock, TX, USA) for a half of the microchannel depicted in Figure 1 is fabricated first by using a two-step lithography process. Next, a PDMS solution (Sylgard 184 elastomer base) and its curing agent are mixed in the weight ratio of 10:1, and then the mixture is poured into the master mold. The workpiece is pumped under vacuum to degas the PDMS and then heated at ${100\;}^{{}^{\circ}}C$ for 60 min in a vacuum drying oven to solidify the PDMS. Subsequently, the PDMS layer is detached from the master mold; each detached PDMS layer is a half of the microchannel. The lower part of the microchannel is stuck on a microscope slide for alignment convenience. Then, the lower part on the microscope slide and the upper part are treated with oxygen plasma. Finally, they are aligned and bonded to finish the fabrication of the whole microchannel. Moreover, a guide channel for an optical fiber delivering light to the microchannel and an observation chamber are also fabricated on the PDMS layers forming the microchannel, as shown in Figure 2.

After the microchannel is fabricated, an F-AM-FC bare fiber adaptor (Newport) is used to connect the light source and an optical fiber. Then, the optical fiber is inserted to the guide channel inside the PDMS layers.

To observe the beam splitting from the top of the microchannel by a microscope connected with a camera, we prepare dyed liquids to be injected into the microchannel by mixing a small amount of Rhodamine B with either calcium chloride solution or deionized water. The fluorescent agent in the dyed liquids serves to visualize the light paths in the microchannel and the observation chamber.

With the silicone tubes attached to the microchannel, we can connect the system to the syringe pumps. To manipulate the L-GRIN in the beam splitter, four streams with different flow rates are required. Thus, we use four syringe pumps to inject calcium chloride solution and deionized water. After the flow achieves steady, we observe the behavior of the flow and the light paths in the L-GRIN beam splitter.

## 5. Results and Discussion

### 5.1. Test of Parameters of Numerical Simulation

**Figure 3:**
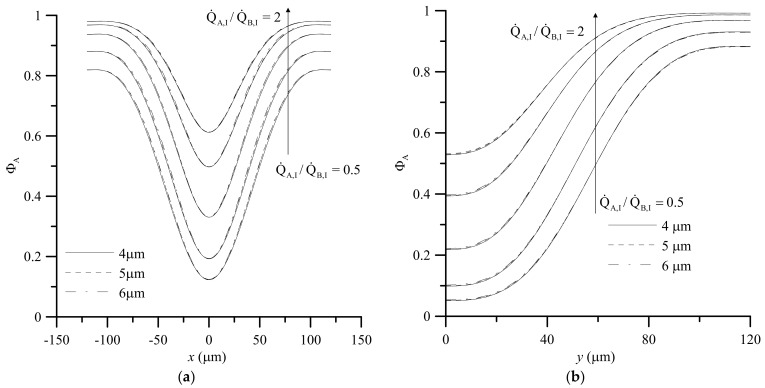
(**a**) The concentration distributions along the centerline (z=0) in x-direction on the half-length plane of main channel, (**b**) those along the centerline (z=0) in y-direction on the plane at $x=\left(W_{I}+L_{S}\right)/2$ of the one-stage beam splitter with h/H=0.5, $L_{I}=240\;\mu m$, $\beta_{I}=0^{{}^{\circ}}$ and different mesh sizes for the cases with various values of ${\dot{Q}}_{A,I}/{\dot{Q}}_{B,I}$ and ${\dot{Q}}_{A,I}+{\dot{Q}}_{B,I}=0.067\;\mu L/s$.

To examine the mesh-size independence, we consider a one-stage beam splitter with ${\;L}_{I}=240\;\mu m$, $\beta_{I}=0^{\circ }$ and h/H=0.5. In this work, we are mainly interested in a mixing flow of calcium chloride solution and deionized water yielding higher variation of refractive index. It is found that the diffusion process dominates in the mixing flow with a small flow rate, such as ${\dot{Q}}_{A,I}+{\dot{Q}}_{B,I}\;=\;0.067\;\mu L/s$. Besides, the diffusion-dominated mixing of working fluids in an optofluidic device leads to an inhomogeneous optical medium with higher variation of refractive index [24]. Thus, we set the total flow rate at 0.067 μL/s and consider the flow rate ratios of the two selected working fluids, ${\dot{Q}}_{A,I}/{\dot{Q}}_{B,I}\;=\;0.067\;\mu L/s$ 0.5, 0.67, 1, 1.5 and 2. Hexahedral meshes are applied to the simulation of the mixing flow in the microchannel. The testing mesh sizes are 6 μm, 5 μm and 4 μm. Comparisons of the concentration distributions along the horizontal centerline in *x*-direction on the half-length plane of the main channel and along the horizontal centerline in *y*-direction on the cross section at $x=\left(W_{I}+L_{S}\right)/2$ of the right inlet branch are shown in Figure 3a,b, respectively. The results show that the concentration distributions with different mesh sizes are mostly overlapping. From the results obtained, we use a mesh size of 5 μm in following simulation.

Before applying the ray tracing codes, we select the number of sampled datum points ($N_{data}$) and the basis vectors employed by the MLS method for the reconstruction of the refractive indices at the Runge-Kutta points and the number of the incident bundles ($N_{b}$) used in the ray tracing procedure for the simulation of light transport. The testing basis vectors include a quadratic, trivariate polynomial expressed as

(18) $${\vec{b}}_{10}\left(\vec{r}\right)=\left[1,x,y,z,xy,yz,zx,x^{2},y^{2},z^{2}\right]$$

and a cubic, trivariate polynomial expressed as

(19) $$\;{\vec{b}}_{20}\left(\vec{r}\right)=\left[1,x,y,z,xy,yz,zx,x^{2},y^{2},z^{2},xyz,x^{2}y,x^{2}z,y^{2}x,y^{2}z,z^{2}x,z^{2}y,x^{3},y^{3},z^{3}\right].$$

We consider the light transport through a two-stage microchannel. Based on the comparison of normalized energy fluxes along *x*-axis with an interval of 5 μm and between z=2.5 μm and z=−2.5 μm on the objective plane obtained by numerical simulation, we select $N_{data}=64$ and ${\vec{b}}_{10}\left(\vec{r}\right)$ in following simulation. The details of comparison are not presented here for the sake of conciseness.

**Figure 4:**
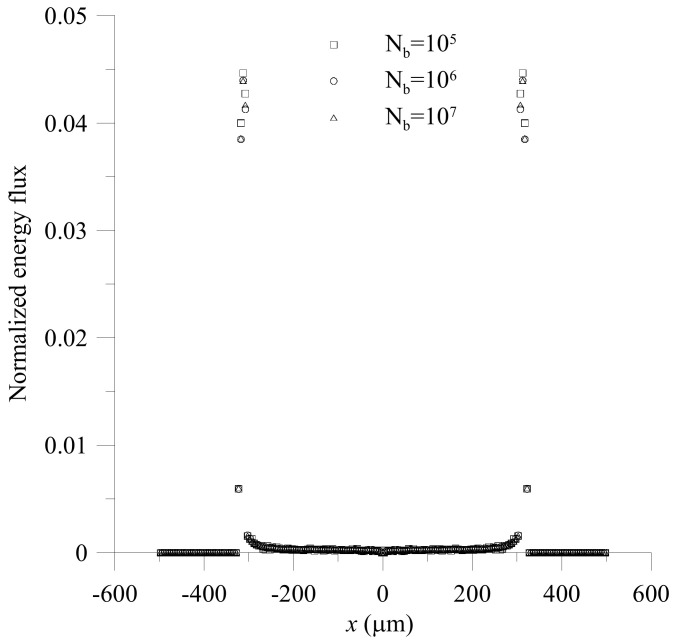
Distributions of normalized energy fluxes on the objective plane of the two-stage beam splitter with h/H=0.25, $L_{I}=240\;\mu m$, $L_{\mathrm{II}}=500\;\mu m$, $\beta_{I}=0^{{}^{\circ}}$, $\beta_{\mathrm{II}}=12^{{}^{\circ}}$, ${\dot{Q}}_{A,I}/{\dot{Q}}_{B,I}=1.5$, ${\dot{Q}}_{A,\mathrm{II}}/{\dot{Q}}_{B,\mathrm{II}}=1$, ${\dot{Q}}_{A,I}+{\dot{Q}}_{B,I}=0.067\;\mu L/s$ and ${\dot{Q}}_{A,\mathrm{II}}+{\dot{Q}}_{B,\mathrm{II}}=0.067\;\mu L/s$ obtained by using a quadratic, trivariate polynomial basis vector, 64 sampling points and different numbers of the incident bundles.

Figure 4 shows the distributions of normalized energy flux along *x*-direction with an interval of 5 μm and between z=2.5 μm and z=−2.5 μm on the objective plane obtained by using ${\vec{b}}_{10}\left(\vec{r}\right)$, $N_{data}=64$, $N_{b}=10^{5}$, $10^{6}$ and $10^{7}$. The CPU times of the simulation using $N_{b}=10^{5}$, $10^{6}$ and $10^{7}$ on i7-4770 CPU @3.40 GHz are 565 s, 5675 s and 56,913 s, respectively. From Figure 4, we can see that the distributions of normalized energy fluxes obtained by using $N_{b}=10^{6}$ and $10^{7}$ are almost overlapping, while the distribution of normalized energy flux obtained by using $N_{b}=10^{5}$ is a little different from the others. Thus, we adopt $N_{b}=10^{6}$ in the cases considered in further discussion.

### 5.2. Comparison of Numerical Results and Experiment Results

**Figure 5:**
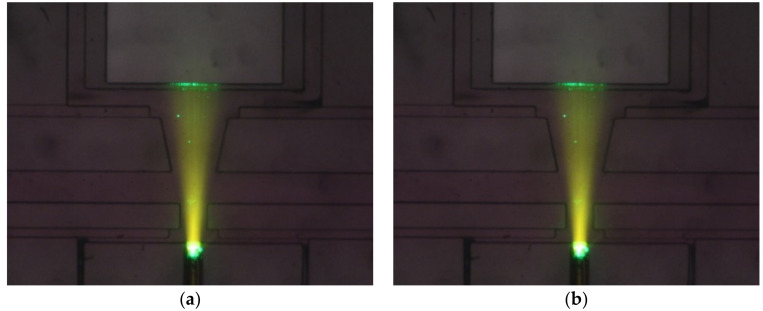
Top views of ray trajectories in the two-stage microchannel with h/H=0.5, $L_{I}=240\;\mu m$, $L_{\mathrm{II}}=500\;\mu m$, $\beta_{I}=0^{{}^{\circ}}$, $\beta_{\mathrm{II}}=12^{{}^{\circ}}$, $W_{A_{I}}=80\;\mu m$, $W_{B_{I}}=40\;\mu m$, $W_{A_{\mathrm{II}}}=150\;\mu m$ and $W_{B_{\mathrm{II}}}=100\;\mu m$ for the cases: (**a**) the microchannel filled with deionized water, (**b**) microchannel filled with Chloride solution injected at inlets $A_{I}$ and $A_{\mathrm{II}}$ and deionized water injected at inlets $B_{I}$ and $B_{\mathrm{II}}$.

**Figure 6:**
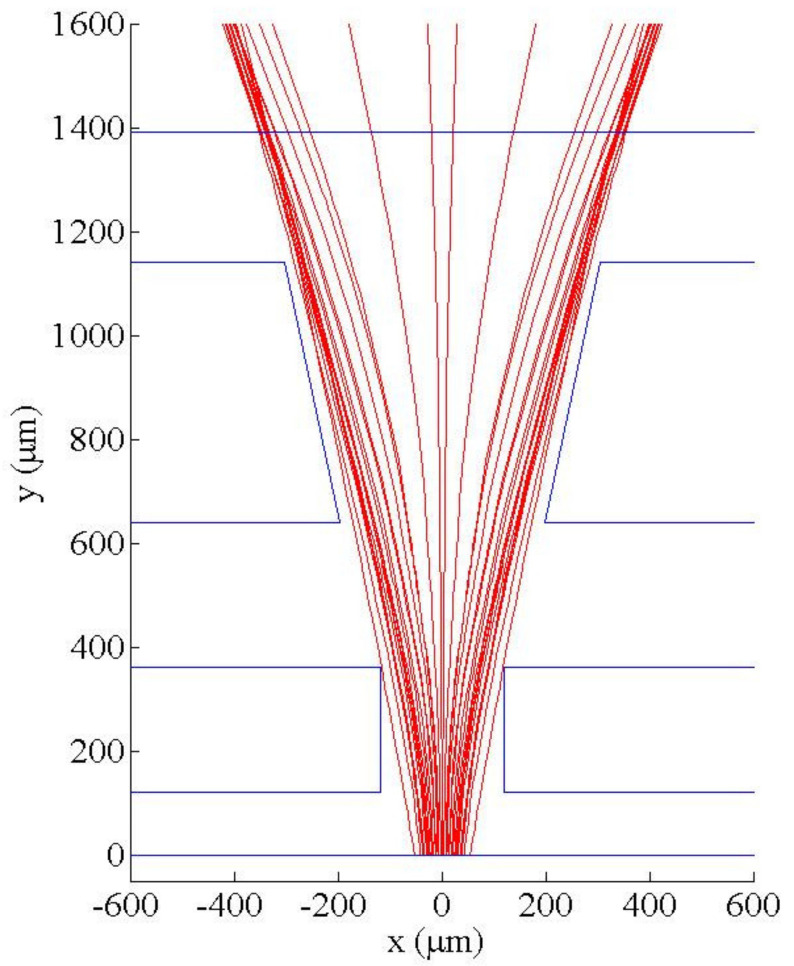
Ray trajectories on the middle horizontal plane of the two-stage beam splitter with h/H=0.5, $L_{I}=240\;\mu m$, $L_{\mathrm{II}}=500\;\mu m$, $\beta_{I}=0^{{}^{\circ}}$, $\beta_{\mathrm{II}}=12^{{}^{\circ}}$, $W_{A_{I}}=80\;\mu m$, $W_{B_{I}}=40\;\mu m$, $W_{A_{\mathrm{II}}}=150\;\mu m$ and $W_{B_{\mathrm{II}}}=100\;\mu m$ for the case with ${\dot{Q}}_{A,I}/{\dot{Q}}_{B,I}=1.5$, ${\dot{Q}}_{A,\mathrm{II}}/{\dot{Q}}_{B,\mathrm{II}}=1$, ${\dot{Q}}_{A,I}+{\dot{Q}}_{B,I}=0.067\;\mu L/s$ and ${\dot{Q}}_{A,\mathrm{II}}+{\dot{Q}}_{B,\mathrm{II}}=0.067\;\mu L/s$.

To show the effectiveness of the proposed beam splitter and to check the validity of numerical simulation, we fabricate and observe the light transport in a two-stage microchannel with h/H=0.5, $L_{I}=240\;\mu m$, $L_{\mathrm{II}}=500\;\mu m$, $\beta_{I}=0^{{}^{\circ}}$, $\beta_{\mathrm{II}}=12^{{}^{\circ}}$, $W_{A_{I}}=80\;\mu m$, $W_{B_{I}}=40\;\mu m$, $W_{A_{\mathrm{II}}}=150\;\mu m$ and $W_{B_{\mathrm{II}}}=100\;\mu m$. Figure 5a shows the top view of ray trajectories in the microchannel filled with deionized water. We can see that the light from source are divergent due to numerical aperture and the rays do not change direction in the medium with uniform refractive index. Then, for comparison purpose, calcium chloride solution is injected at inlets $A_{I}$ and $A_{\mathrm{II}}$ and deionized water is injected at inlets $B_{I}$ and $B_{\mathrm{II}}$, respectively, with ${\dot{Q}}_{A,I}/{\dot{Q}}_{B,I}=1.5$, ${\dot{Q}}_{A,\mathrm{II}}/{\dot{Q}}_{B,\mathrm{II}}=1$, ${\dot{Q}}_{A,I}+{\dot{Q}}_{B,I}=0.067\;\mu L/s$ and ${\dot{Q}}_{A,\mathrm{II}}+{\dot{Q}}_{B,\mathrm{II}}=0.067\;\mu L/s$. The convection-diffusion process of flowing streams in the present two-stage beam splitter yields the gradient of refractive index, which splits the incident light beam into two beams, as shown in Figure 5b. Numerical simulation of the case with the same flow and geometrical parameters is also carried out by using the mesh size of 5 μm, $N_{b}=10^{6}$, $N_{data}=64$ and ${\vec{b}}_{10}\left(\vec{r}\right)$; the ray trajectories on the middle horizontal plane of the two-stage beam splitter obtained by simulation are shown in Figure 6. We can see that the light paths in Figure 5b and those in Figure 6 are in good agreement. Thus, we use the above numerical parameters in following simulation.

### 5.3. Influence of the Geometry of the Beam Splitter

While investigating the influence of the parameters including h/H, $L_{I}$, $L_{\mathrm{II}}$, $\beta_{I}$, $\beta_{\mathrm{II}}$ and the flow rate ratio of the injected cladding fluid and the injected core fluid, we set $W_{I}=240\;\mu m$, $W_{\mathrm{II}}=500\;\mu m$ and H=240 μm. Thus, the value of h/H corresponds to the height of the inlets injected with cladding fluid (h). In the following simulation, other channel widths are found from the relations, $W_{A_{I}}h=W_{B_{I}}H$, ${2(W}_{A_{I}}+W_{B_{I}})=W_{I}$, $W_{A_{\mathrm{II}}}h=W_{B_{\mathrm{II}}}H$, ${2(W}_{A_{\mathrm{II}}}+W_{B_{\mathrm{II}}})=W_{\mathrm{II}}$, $W_{E}=2W_{B_{I}}$ for a one-stage microchannel and $W_{E}=2W_{B_{I}}+2W_{B_{\mathrm{II}}}$ for a two-stage microchannel.

**Figure 7:**
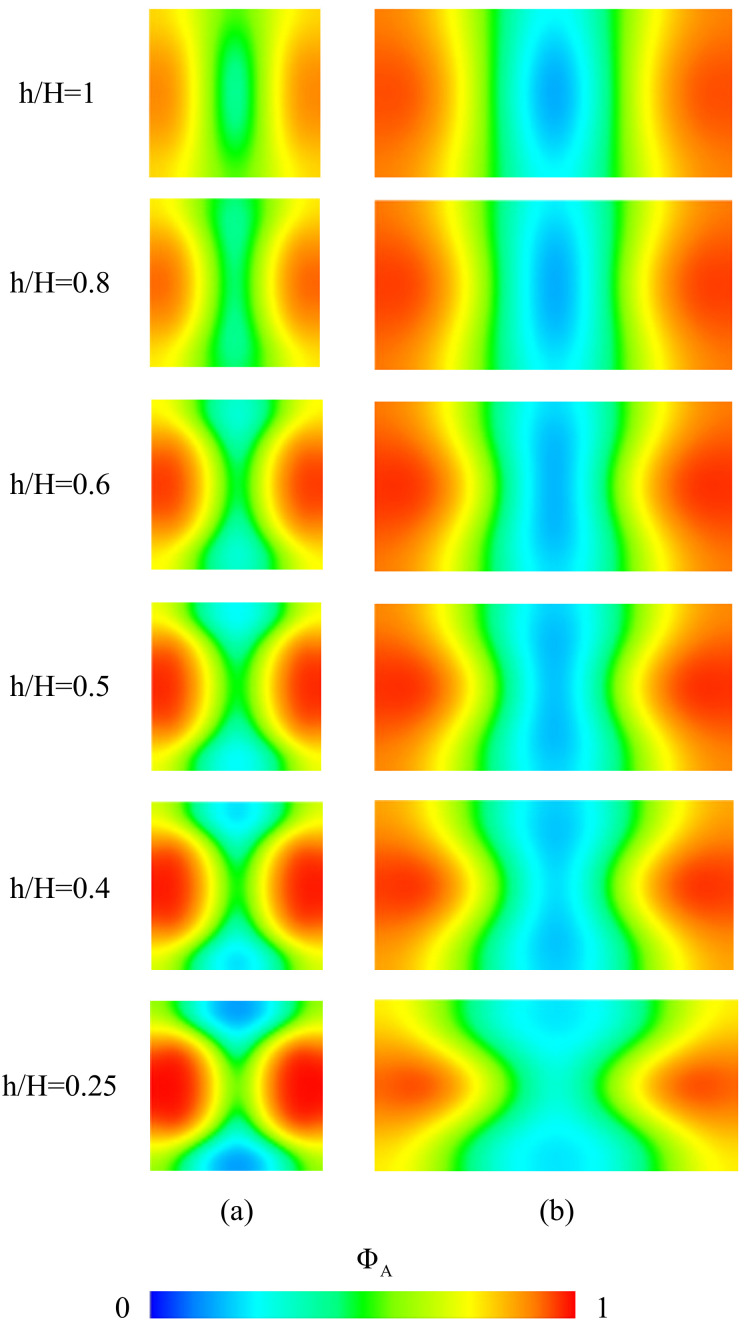
Concentration distributions at the half-length planes of (**a**) the first part; (**b**) the second part of main channels of two-stage beam splitters with $L_{I}=240\;\mu m$, $L_{\mathrm{II}}=500\;\mu m$, $\beta_{I}=0^{{}^{\circ}}$, $\beta_{\mathrm{II}}=12^{{}^{\circ}}$ and various values of h/H for the cases with ${\dot{Q}}_{A,I}/{\dot{Q}}_{B,I}=1.5$, ${\dot{Q}}_{A,\mathrm{II}}/{\dot{Q}}_{B,\mathrm{II}}=1$, ${\dot{Q}}_{A,I}+{\dot{Q}}_{B,I}=0.067\;\mu L/s$ and ${\dot{Q}}_{A,\mathrm{II}}+{\dot{Q}}_{B,\mathrm{II}}=0.067\;\mu L/s$.

**Figure 8:**
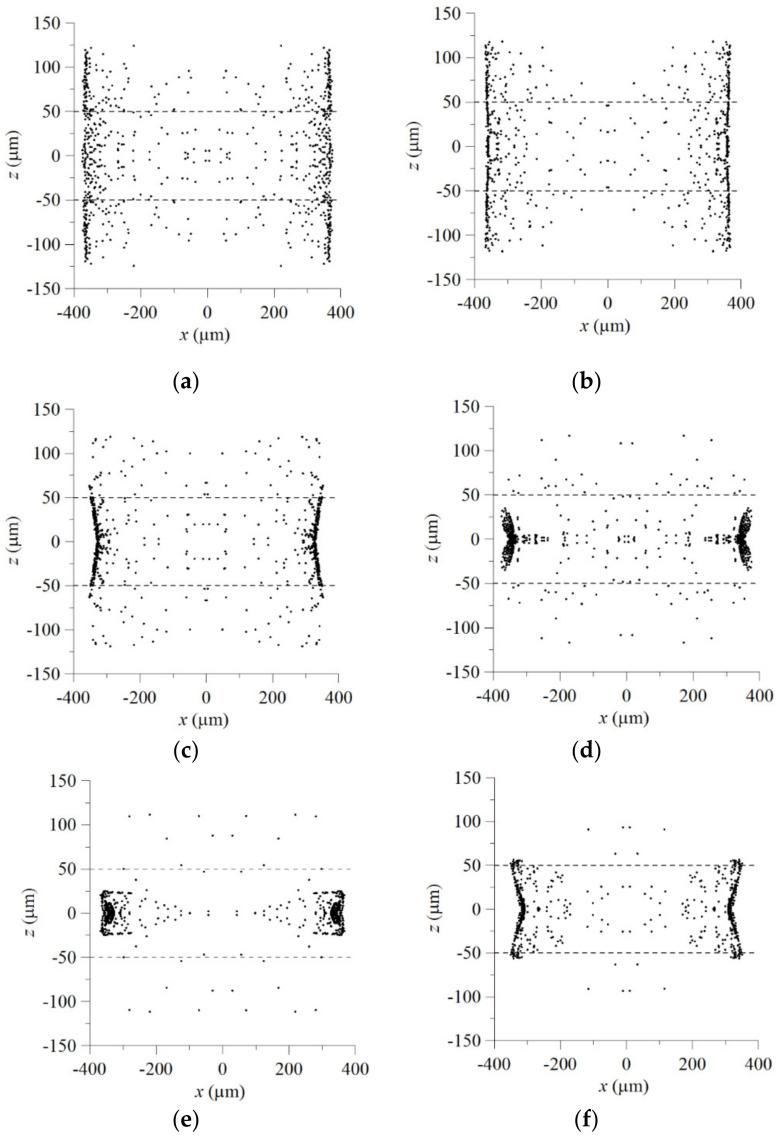
Distributions of bundles on the objective planes of two-stage beam splitters with $L_{I}=240\;\mu m$, $L_{\mathrm{II}}=500\;\mu m$, $\beta_{I}=0^{{}^{\circ}}$, $\beta_{\mathrm{II}}=12^{{}^{\circ}}$ and various values of h/H: (**a**) 1, (**b**) 0.8, (**c**) 0.6, (**d**) 0.5, (**e**) 0.4, (**f**) 0.25 for the cases with ${\dot{Q}}_{A,I}/{\dot{Q}}_{B,I}=1.5$, ${\dot{Q}}_{A,\mathrm{II}}/{\dot{Q}}_{B,\mathrm{II}}=1$, ${\dot{Q}}_{A,I}+{\dot{Q}}_{B,I}=0.067\;\mu L/s$ and ${\dot{Q}}_{A,\mathrm{II}}+{\dot{Q}}_{B,\mathrm{II}}=0.067\;\mu L/s$.

To examine the influence of h/H on the GRIN distribution and the light arriving the objective plane, we examine the concentration distribution and light transport in the mixing fluids through a two-stage beam splitter with $\beta_{I}=0^{{}^{\circ}}$, $L_{I}=240\;\mu m$, $\beta_{\mathrm{II}}=12^{{}^{\circ}}$, $L_{\mathrm{II}}=500\;\mu m$, $L_{s}=100\;\mu m$ and various values of h. Numerical simulation is performed by setting ${\dot{Q}}_{A,I}/{\dot{Q}}_{B,I}=1.5$, ${\dot{Q}}_{A,\mathrm{II}}/{\dot{Q}}_{B,\mathrm{II}}=1$, ${\dot{Q}}_{A,I}+{\dot{Q}}_{B,I}=0.067\;\mu L/s$, ${\dot{Q}}_{A,\mathrm{II}}+{\dot{Q}}_{B,\mathrm{II}}=0.067\;\mu L/s$. The designed inlets of the proposed device yield a 3D concentration distribution in the mixing flow through the main microchannel; the gradient of concentration in either the vertical direction (that is, the *z* direction) or the transverse direction (that is, the *x* direction) can be seen from the cross-section concentration distributions shown in Figure 7a,b. Equation (7) shows that the refractive index of the mixing streams is directly proportional to the concentration. Thus, the GRIN distributions are corresponding to the concentration distributions for the cases considered; consequently, the refractive index of the liquid core flow stream is lower than that of the liquid cladding flow stream and there is more liquid with higher refractive index around the middle horizontal line (that is, z=0) of an *x*-*z* cross section, as shown in Figure 7. Since light prefers to propagate at a lower phase velocity, the transverse distribution of refractive index makes the input light to be separated and transported from the lower refractive index core region (higher phase velocity) to the higher refractive index cladding region (lower phase velocity), as shown in Figure 5b and Figure 6, and the vertical distribution of refractive index of the mixing streams in the microchannel with an appropriate value of h/H makes more light to arrive the higher refractive index cladding region around the middle horizontal line. The effect of the vertical distribution of refractive index can be confirmed by further simulation of light propagation in the mixing streams flowing in the proposed microchannel. Figure 8 shows the distributions of bundles arriving the objective plane for the cases with various values of h/H. It is found that the distributions of bundles arriving the objective plane for the cases with h/H=0.5 or 0.4 are preferable to splitting the beam coupled into the central flow stream from an optical fiber. Thus, we adopt the height ratio of 0.5 in following discussion.

After selecting a preferable height of the inlets injected with cladding fluid, we investigate the influence of the geometry parameters of the main channel, $L_{I}$, $\beta_{I}$, $L_{\mathrm{II}}$ and $\beta_{\mathrm{II}}$. To simplify analysis, we consider first the one-stage microchannel corresponding to the first part of the proposed two-stage microchannel to find the preferable values of $L_{I}$ and $\beta_{I}$. Numerical simulation is performed by setting h/H=0.5,${\dot{Q}}_{A,I}/{\dot{Q}}_{B,I}=1.5$ and ${\dot{Q}}_{A,I}+{\dot{Q}}_{A,I}=$0.067 μL/s. Since the incident rays are divergent and bending to both sides of *y*-axis in the device, the rays tend to intersect the side walls of main channel with $\beta_{I}<0^{{}^{\circ}}$. Thus, the cases with $\beta_{I}<0^{{}^{\circ}}$ are not considered in this work.

**Figure 9:**
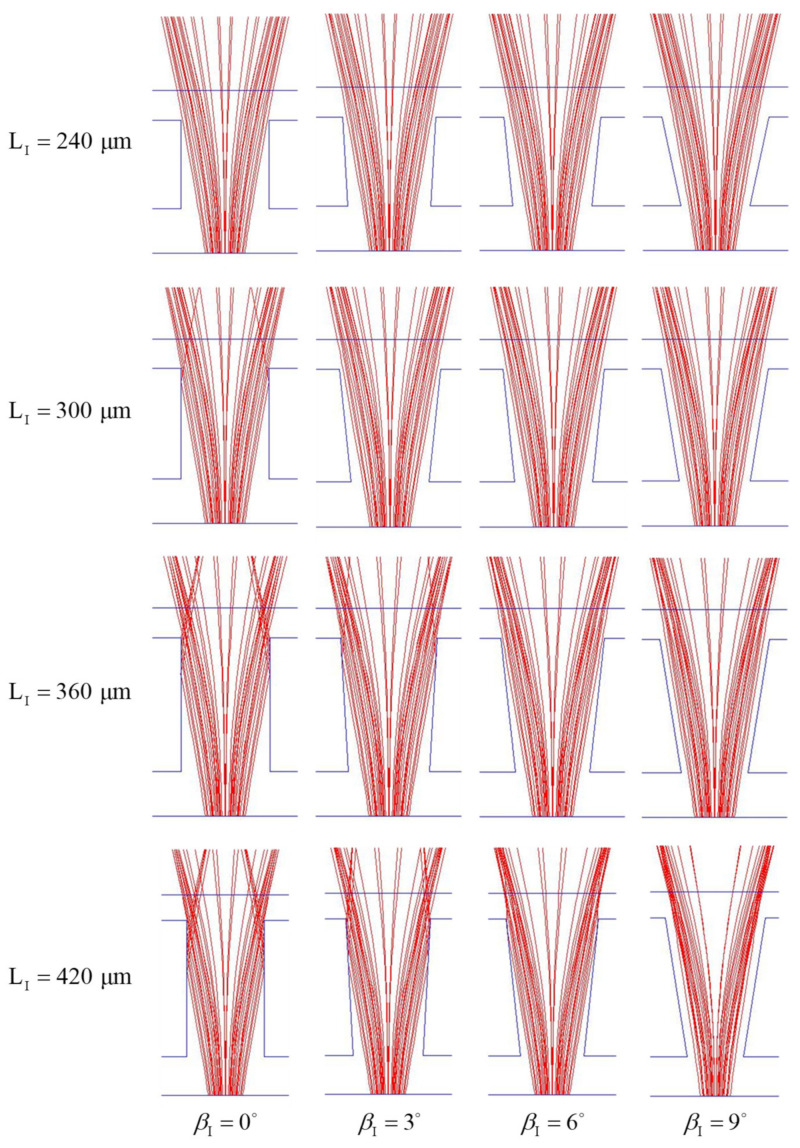
Ray trajectories projected on the middle horizontal plane (z=0) of one-stage beam splitters with h/H=0.5, $L_{I}=240$, 300, 360, 420 μm, $\beta_{I}=0^{{}^{\circ}}$, $3^{{}^{\circ}}$, $6^{{}^{\circ}}$, $9^{{}^{\circ}}$ for the cases with ${\dot{Q}}_{A,I}/{\dot{Q}}_{B,I}=1.5$ and ${\dot{Q}}_{A,I}+{\dot{Q}}_{B,I}=0.067\;\mu L/s$.

The ray trajectories of the cases with various combinations of $L_{I}$ and $\beta_{I}$ projected on the middle horizontal plane are shown in Figure 9 and the $E_{ob}$, the $E_{\pm 50\mu m}$ and the $\theta_{s}$ of those cases are listed in Table 1. From Figure 9, it is readily to see that more rays intersect with the sidewalls of a channel with $\beta_{I}=0^{{}^{\circ}}$, because the rays diverge with propagation. Thus, energy arriving the objective plane decreases with the increase of $L_{I}$ for the cases with $\beta_{I}=0^{{}^{\circ}}$, as shown in Table 1. On the other hand, the $E_{\pm 50\mu m}$ and the $E_{ob}$ increases with the increase of $L_{I}$ for the cases with $\beta_{I}=6^{{}^{\circ}}\;\mathrm{and}\;9^{{}^{\circ}}$, because the divergent sidewalls of the main channel and the GRIN distribution in flowing streams yield less intersection of the sidewalls and the rays and make more energy bundles arrive the zone between z=50 μm and −50 μm on the objective plane. From Table 1, we find the following trends. (i) The influence of $L_{I}$ and $\beta_{I}$ on the $E_{ob}$ is stronger than that of $L_{I}$ and $\beta_{I}$ on the $\theta_{s}$. (ii) The average of $E_{ob}$ over $\beta_{I}$ increases with the increase of $L_{I}$. (iii) The influence of $\beta_{I}$ on the $E_{ob}$ increases with the increase of $L_{I}$. (iv) For the cases with $L_{I}=$ 300, 360 or 420 μm, the values of $E_{\pm 50\mu m}$ and $E_{ob}$ increase with the increase of $\beta_{I}$. (v) In general, the $E_{ob}$ of a case with relatively larger $L_{I}$ and $\beta_{I}$ is larger. Therefore, we select $L_{I\;}=420\;\mu m$ and $\beta_{I}=9^{{}^{\circ}}$ as the working parameters of the first part of the microchannel for further parameter study, because using a microchannel with $L_{I}=420\;\mu m$ and $\beta_{I}=9^{{}^{\circ}}$ can yield a high $E_{ob}$ and a medium $\theta_{s}$.

**Figure 10:**
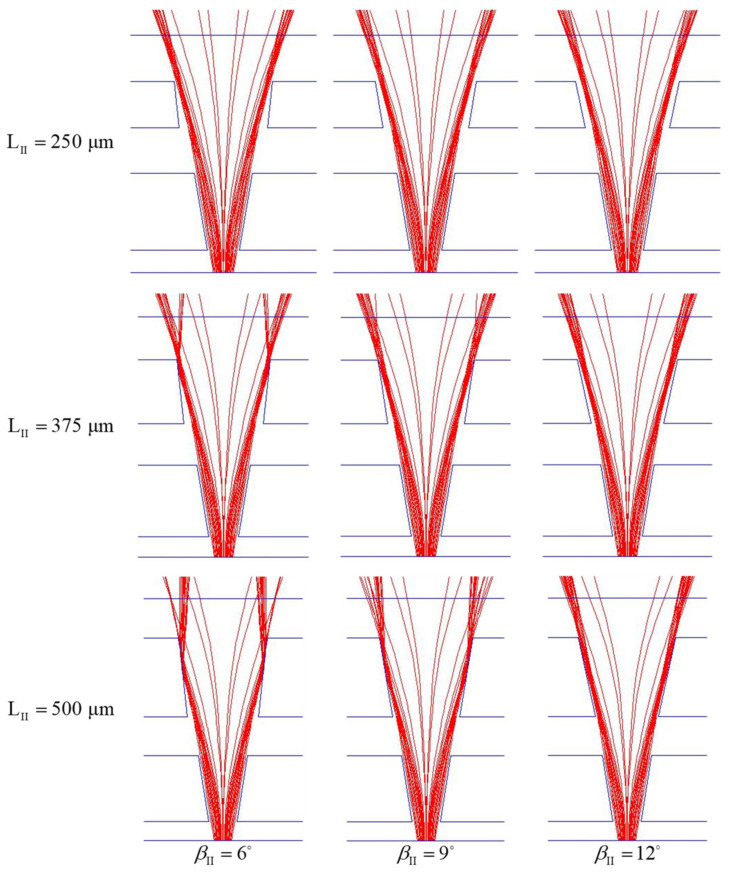
Ray trajectories on the middle horizontal plane (z=0 ) of two-stage beam splitters with h/H=0.5, $L_{I}=420\;\mu m$, $\beta_{I}=9^{{}^{\circ}}$, $L_{\mathrm{II}}=250$, 350, 500 μm and $\beta_{\mathrm{II}}=6^{{}^{\circ}},$ $9^{{}^{\circ}}$, $12^{{}^{\circ}}$, respectively, for the cases with ${\dot{Q}}_{A,I}/{\dot{Q}}_{B,I}=1.5$, ${\dot{Q}}_{A,\mathrm{II}}/{\dot{Q}}_{B,\mathrm{II}}=1$, ${\dot{Q}}_{A,I}+{\dot{Q}}_{B,I}=0.067\;\mu L/s$ and ${\dot{Q}}_{A,\mathrm{II}}+{\dot{Q}}_{B,\mathrm{II}}=0.067\;\mu L/s$.

Next, based on the analysis of the previous paragraph, we investigate the effects of $L_{\mathrm{II}}$ and $\beta_{\mathrm{II}}$ on the performance of beam splitting in the two-stage beam splitter with $L_{I}=420\;\mu m$ and $\beta_{I}=9^{{}^{\circ}}$. Since more rays entering the second part of the two-stage microchannel have larger bending angles, we consider main channels with $L_{\mathrm{II}}=250,$ 375, 500 μm, $\beta_{\mathrm{II}}=6^{{}^{\circ}},$ $9^{{}^{\circ}}$, $12^{{}^{\circ}}$, and set the flow rate ratio (${\dot{Q}}_{A,\mathrm{II}}/{\dot{Q}}_{B,\mathrm{II}}$) to be 1 and the total flow rate (${\dot{Q}}_{A,\mathrm{II}}+{\dot{Q}}_{B,\mathrm{II}}$) to be 0.067 μL/s. From ray trajectories projected on the horizontal plane at z=0 shown in Figure 10, we can see that the number of the rays intersecting the channel sidewalls increase with the increase of $L_{\mathrm{II}}$ for the cases with a smaller $\beta_{\mathrm{II}}$ ($6^{{}^{\circ}}$ or $9^{{}^{\circ}}$). The intersection of the rays and the sidewalls reduces the light energy arriving the objective plane. Thus, when $\beta_{\mathrm{II}}=6^{{}^{\circ}}$ or $9^{{}^{\circ}}$, the $E_{\pm 50\mu m}$ and $E_{ob}$ decrease with the increase of $L_{\mathrm{II}}$, as shown in Table 2. Moreover, when $\beta_{\mathrm{II}}=12^{{}^{\circ}}$, both $E_{\pm 50\mu m}$ and $E_{ob}$ of the case with $L_{\mathrm{II}}=375\;\mu m$ are maximum among the three cases with $\beta_{\mathrm{II}}=12^{{}^{\circ}}$. From Table 2, we also see that the $\theta_{s}$ of this case is medium. Similar to the trend found from the flow in the one-stage beam splitter, the influence of $L_{\mathrm{II}}$ and $\beta_{\mathrm{II}}$ on $E_{ob}$ is stronger than that of $L_{\mathrm{II}}$ and $\beta_{\mathrm{II}}$ on $\theta_{s}$, as shown in Table 2. Therefore, we select $L_{\mathrm{II}}=375\;\mu m$ and $\beta_{\mathrm{II}}=12^{o}$ as the working parameters of the second part of the two-stage beam splitter. Using the two-stage beam splitter with $L_{\mathrm{II}}=375\;\mu m$ and $\beta_{\mathrm{II}}=12^{{}^{\circ}}$ enlarges both of $E_{ob}$ and $\theta_{s}$.

### 5.4. Effect of the Ratio of Flow Rates

In addition to geometry parameters, the ratio of flow rates is the other important parameter, which can be tuned to enhance the performance of the proposed beam splitter. Here, we first investigate the effects of the flow rate ratio in the one-stage beam splitter considered in Section 5.3. Numerical simulation is carried out to calculate the $E_{ob}$ and $\theta_{s}$ of the cases with ${\dot{Q}}_{A,I}/{\dot{Q}}_{B,I}=$0.33, 0.5, 0.67, 1, 1.5, 2 and ${\dot{Q}}_{A,I}+{\dot{Q}}_{B,I}=0.067\;\mu L/s$. From the results listed in Table 3, we can see that the increase of the flow rate ratio leads to the decrease of $\theta_{s}$ and the increase of $E_{ob}$, until $E_{ob}$ reaches a maximum value as ${\dot{Q}}_{A,I}/{\dot{Q}}_{B,I}=1.5$. When we further increase the flow rate ratio, the corresponding decrease of $E_{ob}$ is small and the decrease of $\theta_{s}$ is large. Taking both variations of $E_{ob}$ and $\theta_{s}$ into account, we select ${\dot{Q}}_{A,I}/{\dot{Q}}_{B,I}=1.5$ for the first part of the two-stage beam splitter.

**Figure 11:**
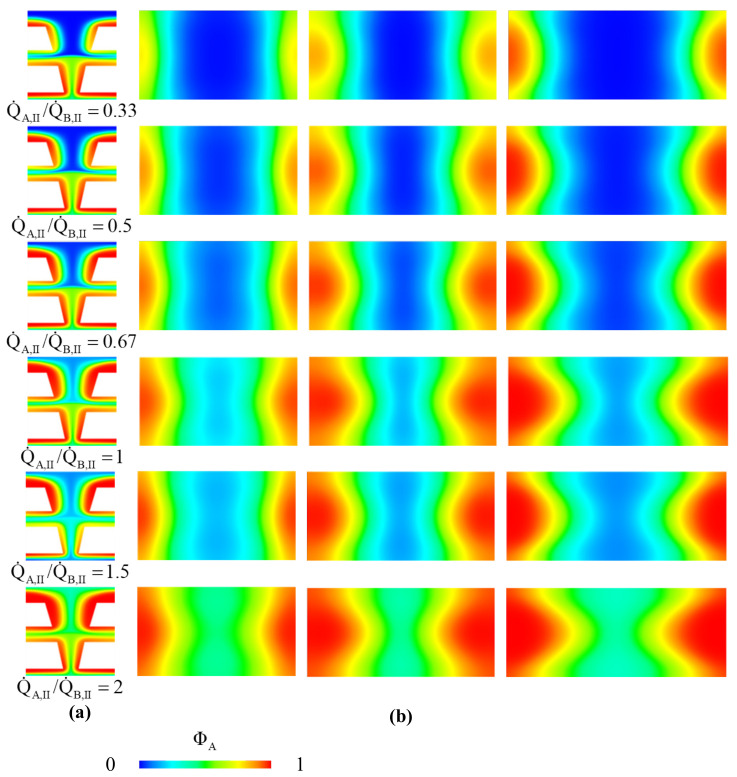
The concentration distributions on (**a**) horizontal plane at z=0 and (**b**) the vertical cross sections at $y=y_{1}=W_{C_{I}}+L_{I}+W_{E}$, $y=y_{1}+L_{\mathrm{II}}/2$ and $y=y_{1}+L_{\mathrm{II}}$ of the main channel for the cases with h/H=0.5, $L_{I}=420\;\mu m$, $\beta_{I}=9^{{}^{\circ}}$, $L_{\mathrm{II}}=375\;\mu m$ and $\beta_{\mathrm{II}}=12^{{}^{\circ}}$, ${\dot{Q}}_{A,I}/{\dot{Q}}_{B,I}=1.5$, various values of ${\dot{Q}}_{A,\mathrm{II}}/{\dot{Q}}_{B,\mathrm{II}}$, ${\dot{Q}}_{A,I}+{\dot{Q}}_{B,I}=0.067\;\mu L/s$ and ${\dot{Q}}_{A,\mathrm{II}}+{\dot{Q}}_{B,\mathrm{II}}=0.067\;\mu L/s$.

Next, setting ${\dot{Q}}_{A,I}/{\dot{Q}}_{B,I}=1.5$, we examine the influence of the flow rate ratio of the second part of microchannel. The $E_{ob}$ and $\theta_{s}$ of the cases with ${\dot{Q}}_{A,\mathrm{II}}/{\dot{Q}}_{B,\mathrm{II}}=$0.33, 0.5, 0.67, 1, 1.5, 2 and ${\dot{Q}}_{A,\mathrm{II}}+{\dot{Q}}_{B,\mathrm{II}}=0.067\;\mu L/s$ are listed in Table 4. The concentration distributions on the horizontal plane (z=0) and the vertical cross sections at $y=y_{1}=W_{C_{I}}+L_{I}+W_{E}$, $y=y_{1}+L_{\mathrm{II}}/2$ and $y=y_{1}+L_{\mathrm{II}}$ of the main channel are shown in Figure 11. From Equation (7), we know that the concentration distribution corresponds to the GRIN distribution. From Table 4, we can see that the increase of ${\dot{Q}}_{A,\mathrm{II}}/{\dot{Q}}_{B,\mathrm{II}}$ leads to the decrease of $\theta_{s}$. This is because the diffusion zones between the core flow stream and the cladding flow stream get close and the gradient of refractive index decreases with the increase of ${\dot{Q}}_{A,\mathrm{II}}/{\dot{Q}}_{B,\mathrm{II}}$, as shown in Figure 11. Such a GRIN distribution reduces the bend angle of the light refracted into the two cladding flow streams. Moreover, from Table 4, we can see that the increase of ${\dot{Q}}_{A,\mathrm{II}}/{\dot{Q}}_{B,\mathrm{II}}$ from 0.33 leads to the increase of $E_{ob}$ until ${\dot{Q}}_{A,\mathrm{II}}/{\dot{Q}}_{B,\mathrm{II}}=1$. Further increase of the flow rate ratio makes $E_{ob}$ decrease obviously and $\theta_{s}$ decrease a little. Therefore, we select ${\dot{Q}}_{A,\mathrm{II}}/{\dot{Q}}_{B,\mathrm{II}}=1$ for the second part of the two-stage beam splitter.

## 6. Conclusions

An L-GRIN beam splitter with a designed microchannel is proposed to enlarge the split angle and to reduce the light loss of the device. The designed microchannel includes a two-stage main channel with divergent side walls and two pairs of inlet channels, and the height of its inlets injected with cladding fluid is set to be less than the main-channel height. The results of simulation and experiment show the effectiveness of the beam splitter. Some concluding remarks can be drawn from the results obtained. (i) A split angle larger than $36^{{}^{\circ}}$ are achieved by selecting preferable geometrical parameters and tuning the flow rate ratio. (ii) Setting h/H= 0.5 or 0.4 makes more light arrive the optical fibers coupled with the L-GRIN beam splitter, and so enhances the transmission efficiency of the device. (iii) The improvement of the transmission of the device by setting h/H= 0.5 or 0.4 increases with the increase of length of the main channel with divergent side walls. (iv) Appropriate combination of divergent angle of side walls and lengths of the two parts of main channel may increases the split angle. (v) The increase of the flow rate ratio leads to the decrease of $\theta_{s}$ and the increase of $E_{ob}$, until $E_{ob}$ reaches the maximum. Consequently, the present L-GRIN beam splitter may have potential applications in integrated optofluidic communication systems.

## Figures and Tables

**Table 1:** Effects of main channel length and sidewall divergent angle on the $E_{\pm 50\mu m}$, the $E_{ob}$ and the $\theta_{s}$ of one-stage beam splitters for the cases with h/H=0.5, ${\dot{Q}}_{A,I}/{\dot{Q}}_{B,I}=1.5$ and ${\dot{Q}}_{A,I}+{\dot{Q}}_{B,I}=0.067\;\mu L/s$.

$L_{I}$	$\beta_{I}$	$E_{\pm 50\mu m}$	$E_{ob}$	$\theta_{s}$
240 μm	$0^{{}^{\circ}}$	0.6128	0.4626	$26.27^{{}^{\circ}}$
	$3^{{}^{\circ}}$	0.6118	0.4633	$26.09^{{}^{\circ}}$
	$6^{{}^{\circ}}$	0.6137	0.4696	$25.72^{{}^{\circ}}$
	$9^{{}^{\circ}}$	0.5532	0.4327	$24.63^{{}^{\circ}}$
300 μm	$0^{{}^{\circ}}$	0.5713	0.4432	$25.91^{{}^{\circ}}$
	$3^{{}^{\circ}}$	0.6110	0.4738	$25.54^{{}^{\circ}}$
	$6^{{}^{\circ}}$	0.6207	0.4885	$25.36^{{}^{\circ}}$
	$9^{{}^{\circ}}$	0.6201	0.4965	$25.16^{{}^{\circ}}$
360 μm	$0^{{}^{\circ}}$	0.5228	0.4195	$28.46^{{}^{\circ}}$
	$3^{{}^{\circ}}$	0.5868	0.4575	$27.89^{{}^{\circ}}$
	$6^{{}^{\circ}}$	0.6358	0.5174	$26.09^{{}^{\circ}}$
	$9^{{}^{\circ}}$	0.6369	0.5287	$25.36^{{}^{\circ}}$
420 μm	$0^{{}^{\circ}}$	0.4684	0.3821	$28.07^{{}^{\circ}}$
	$3^{o}$	0.5585	0.4613	$26.81^{{}^{\circ}}$
	$6^{{}^{\circ}}$	0.6378	0.5370	$26.63^{{}^{\circ}}$
	$9^{{}^{\circ}}$	0.6592	0.5698	$26.45^{{}^{\circ}}$

**Table 2:** Effects of the main channel length ($L_{\mathrm{II}}$ ) and the divergent angle ($\beta_{\mathrm{II}}$ ) on the $E_{\pm 50\mu m}$, the $E_{ob}$ and the $\theta_{s}$ of two-stage beam splitters with h/H=0.5, $L_{I}=420\;\mu m$ and $\beta_{I}=9^{{}^{\circ}}$ for the case with ${\dot{Q}}_{A,I}/{\dot{Q}}_{B,I}=1.5$, ${\dot{Q}}_{A,\mathrm{II}}/{\dot{Q}}_{B,\mathrm{II}}=1$, ${\dot{Q}}_{A,I}+{\dot{Q}}_{B,I}=0.067\;\mu L/s$ and ${\dot{Q}}_{A,\mathrm{II}}+{\dot{Q}}_{B,\mathrm{II}}=0.067\;\mu L/s$.

$L_{\mathrm{II}}$	$\beta_{\mathrm{II}}$	$E_{\pm 50\mu m}$	$E_{ob}$	$\theta_{s}$
250 μm	$6^{{}^{\circ}}$	0.9504	0.8639	$35.84^{{}^{\circ}}$
	$9^{{}^{\circ}}$	0.9522	0.8790	$32.87^{{}^{\circ}}$
	$12^{{}^{\circ}}$	0.9517	0.8805	$34.27^{{}^{\circ}}$
375 μm	$6^{{}^{\circ}}$	0.7936	0.7278	$40.27^{{}^{\circ}}$
	$9^{{}^{\circ}}$	0.8974	0.8303	$39.26^{{}^{\circ}}$
	$12^{{}^{\circ}}$	0.9537	0.8856	$36.87^{{}^{\circ}}$
	$6^{{}^{\circ}}$	0.3034	0.2478	$39.77^{{}^{\circ}}$
500 μm	$9^{{}^{\circ}}$	0.5092	0.4417	$40.95^{{}^{\circ}}$
	$12^{{}^{\circ}}$	0.8370	0.7784	$42.94^{{}^{\circ}}$

**Table 3:** Effects of flow rate ratio (${\dot{Q}}_{A,I}/{\dot{Q}}_{B,I}$ ) on the $E_{ob}$ and the $\theta_{s}$ of a one-stage beam splitter with h/H=0.5, $L_{I}=420\;\mu m$ and $\beta_{I}=9^{{}^{\circ}}$ for the cases with ${\dot{Q}}_{A,I}+{\dot{Q}}_{A,I}=0.067\;\mu L/s$.

${\dot{Q}}_{A,I}/{\dot{Q}}_{B,I}$	$E_{ob}$	$\theta_{s}$
0.33	0.3776	$31.28^{{}^{\circ}}$
0.50	0.4493	$30.75^{{}^{\circ}}$
0.67	0.4998	$30.57^{{}^{\circ}}$
1.00	0.5538	$27.17^{{}^{\circ}}$
1.50	0.5698	$26.45^{{}^{\circ}}$
2.00	0.5512	$22.80^{{}^{\circ}}$

**Table 4:** Effects of flow rate ratio (${\dot{Q}}_{A,\mathrm{II}}/{\dot{Q}}_{B,\mathrm{II}}$) on the $E_{ob}$ and the $\theta_{s}$ of a two-stage beam splitter with h/H=0.5, $L_{I}=420\;\mu m$, $\beta_{I}=9^{{}^{\circ}}$, $L_{\mathrm{II}}=375\;\mu m$ and $\beta_{\mathrm{II}}=12^{{}^{\circ}}$ for the cases with ${\dot{Q}}_{A,I}/{\dot{Q}}_{B,I}=1.5$, ${\dot{Q}}_{A,I}+{\dot{Q}}_{B,I}=0.067\;\mu L/s$ and ${\dot{Q}}_{A,\mathrm{II}}+{\dot{Q}}_{B,\mathrm{II}}=0.067\;\mu L/s$.

${\dot{Q}}_{A,II}/{\dot{Q}}_{B,II}$	$E_{ob}$	$\theta_{s}$
0.33	0.7934	$41.78^{{}^{\circ}}$
0.50	0.8237	$41.78^{{}^{\circ}}$
0.67	0.8295	$40.27^{{}^{\circ}}$
1.00	0.8856	$36.87^{{}^{\circ}}$
1.50	0.8351	$36.87^{{}^{\circ}}$
2.00	0.8282	$36.00^{{}^{\circ}}$

## Data Availability

Not applicable.

## References

[1] Song C., Tan S.H. (2017). A perspective on the rise of optofluidics and the future. Micromachines.

[2] Erickson D., Sinton D., Psaltis D. (2011). Optofluidics for energy applications. Nat. Photonics.

[3] Lei L., Wang N., Zhang X.M., Tai Q., Tsai D.P., Chan H.L.W. (2010). Optofluidic planar reactors for photocatalytic water treatment using solar energy. Biomicrofluidics.

[4] Fan X.D., White I.M. (2011). Optofluidic microsystems for chemical and biological analysis. Nat. Photonics.

[5] Myers F.B., Lee L.P. (2008). Innovations in optical microfluidic technologies for point-of-care diagnostics. Lab Chip.

[6] Weber E., Vellekoop M.J. (2012). Optofluidic micro-sensors for the determination of liquid concentrations. Lab Chip.

[7] Nguyen N.-T. (2010). Micro-optofluidic lenses: A review. Biomicrofluidics.

[8] Tang X., Liang S., Li R. (2015). Design for controllable optofluidic beam splitter. Photonics Nanostruct. Fundam. Appl..

[9] Seow Y.C., Liu A.Q., Chin L.K., Li X.C., Huang H.J., Cheng T.H., Zhou X.Q. (2008). Different curvatures of tunable liquid microlens via the control of laminar flow rate. Appl. Phys. Lett..

[10] Shi J., Stratton Z., Lin S.C.S., Huang H., Huang T.J. (2009). Tunable optofluidic microlens through active pressure control of an air–liquid interface. Microfluid. Nanofluid..

[11] Mao X., Waldeisen J.R., Juluri B.K., Huang T.J. (2007). Hydrodynamically tunable optofluidic cylindrical microlens. Lab Chip.

[12] Mao X., Lin S.C.S., Lapsley M.I., Shi J., Juluri B.K., Huang T.J. (2009). Tunable liquid gradient refractive index (L-GRIN) lens with two degrees of freedom. Lab Chip.

[13] Mishra K., Murade C., Carreel B., Roghair I., Oh J.M., Manukyan G., van den Ende D., Mugele F. (2014). Optofluidic lens with tunable focal length and asphericity. Sci. Rep..

[14] Le Z., Sun Y., Du Y. (2015). Liquid gradient refractive index microlens for dynamically adjusting the beam focusing. Micromachines.

[15] Miccio L., Memmolo P., Merola F., Netti P.A., Ferraro P. (2015). Red blood cell as an adaptive optofluidic microlens. Nat. Commun..

[16] Hamilton E.S., Ganjalizadeh V., Wright J.G., Schmidt H., Hawkins A.R. (2020). 3D hydrodynamic focusing in microscale optofluidic channels formed with a single sacrificial layer. Micromachines.

[17] Song C., Nguyen N.T., Asundi A.K., Tan S.H. (2010). Tunable micro-optofluidic prism based on liquid-core liquid-cladding configuration. Opt. Lett..

[18] Xiong S., Liu A.Q., Chin L.K., Yang Y. (2011). An optofluidic prism tuned by two laminar flows. Lab Chip.

[19] Wolfe D.B., Conroy R.S., Garstecki P., Mayers B.T., Fischbach M.A., Paul K.E., Prentiss M., Whitesides G.M. (2004). Dynamic control of liquid-core/liquid-cladding optical waveguides. Proc. Natl. Acad. Sci. USA.

[20] Wolfe D.B., Vezenov D.V., Mayers B.T., Whitesides G.M., Conroy R.S., Prentiss M.G. (2005). Diffusion-controlled optical elements for optofluidics. Appl. Phys. Lett..

[21] Schmidt H., Hawkins A.R. (2008). Optofluidic waveguides: I. Concepts and implementations. Microfluid. Nanofluid..

[22] Schmidt H., Hawkins A.R. (2007). Optofluidic waveguides: II. Fabrication and structures. Microfluid. Nanofluid..

[23] Yang Y., Liu A.Q., Chin L.K., Zhang X.M., Tsai D.P., Lin C.L., Lu C., Wang G.P., Zheludev N.I. (2012). Optofluidic waveguide as a transformation optics device for lightwave bending and manipulation. Nat. Commun..

[24] Yang Y., Chin L.K., Tsai J.M., Tsai D.P., Zheludev N.I., Liu A.Q. (2012). Transformation optofluidics for large-angle light bending and tuning. Lab Chip.

[25] Li L., Zhu X.Q., Liang L., Zuo Y.F., Xu Y.S., Yang Y., Yuan Y.J., Huang Q.Q. (2016). Switchable 3D optofluidic Y-branch waveguides tuned by Dean flows. Sci. Rep..

[26] David R.L. (2005). CRC Handbook of Chemistry and Physics.

[27] Lyons P.A., Riley J.F. (1954). Diffusion coefficients for aqueous solutions of calcium chloride and cesium chloride at 25 °C. J. Am. Chem. Soc..

[28] Mawlud S.Q., Muhamad N.Q. (2012). Theoretical and experiment study of a numerical aperture for multimode PCS fiber optics using an imaging technique. Chin. Phys. Lett..

[29] Born M., Wolf E. (1999). Principles of Optics: Electromagnetic Theory of Propagation, Interference and Diffraction of Light.

[30] Sharma A., Kumar D.V., Ghatak A.K. (1982). Tracing rays through graded-index media: A new method. Appl. Opt..

[31] Dormand J.R., Prince P.J. (1980). A family of embedded Runge-Kutta formulae. J. Comput. Appl. Math..

[32] Nealen A. (2004). An as-short-as-possible introduction to the least squares, weighted least squares and moving least squares methods for scattered data approximation and interpolation. Comput. Meth. Appl. Mech. Eng..

[33] Kuo M.-Y., Wu C.-Y., Hsu K.-C., Chang C.-Y., Jiang W. (2019). Numerical investigation of high-Peclet-number mixing in periodically-curved microchannel with strong curvature. Heat Transf. Eng..

[34] Sharma A., Ghatak A.K. (1986). Ray tracing in gradient-index lenses: Computation of ray-surface intersection. Appl. Opt..

